# Correction: Positive impact of a faecal-based screening programme on colorectal cancer mortality risk

**DOI:** 10.1371/journal.pone.0258077

**Published:** 2021-09-27

**Authors:** The PLOS ONE Staff

The funding statement is incorrect. The publisher apologizes for the error. The correct statement is: This study was partially cofounded by the Carlos III Health Institute, the European Regional Development Fund–a way to build Europe- (PI2/00992, PI16/00588), by the Department of Universities and Research (2017 SGR 723, 2017 SGR 1283) of the Government of Catalonia, and the Spanish Association Against Cancer (AECC) Scientific Foundation.
