# Supplementary material for: Positive impact of a faecal-based screening programme on colorectal cancer mortality risk
Source: PLoS One. 2021 Jun 30;16(6):e0253369. doi: 10.1371/journal.pone.0253369 (PMC8244848; doi:10.1371/journal.pone.0253369)

**S1 File: Positive impact of a faecal-based screening programme on colorectal cancer mortality risk**

Gemma Ibáñez-Sanz, Núria Milà; Carmen Vidal; Judith Rocamora; Víctor Moreno; Rebeca Sanz-Pamplona; Montse Garcia on behalf of the MSIC-SC research group

**S1 Table. Characteristics of g-FOBT participants with interval cancer defined as those diagnosed ≤30 months**

|  | **g-FOBT** | | |
| --- | --- | --- | --- |
|  | **Screening** | **Interval** | **p-value** |
|  | ***n=75*** | ***n=62*** |  |
| Sex |  |  | 1.000 |
| Female | 92 (34.7%) | 44 (34.9%) |  |
| Male | 173 (65.3%) | 82 (65.1%) |  |
| Age at diagnosis (median, years) | 61.9 (5.47) | 63.1 (5.18) | 0.041 |
| Socioeconomic Score |  |  | 0.533 |
| 0-39 (least deprived) | 10 (3.77%) | 2 (1.59%) |  |
| 39-51 | 80 (30.2%) | 37 (29.4%) |  |
| 52-100 (most deprived) | 175 (66.0%) | 87 (69.0%) |  |
| Last participation |  |  | <0.001 |
| Initial | 191 (72.1%) | 53 (42.1%) |  |
| Successive | 74 (27.9%) | 73 (57.9%) |  |
| Tumour Location |  |  | 0.001 |
| Distal | 140 (52.8%) | 43 (34.1%) |  |
| Proximal | 61 (23.0%) | 33 (26.2%) |  |
| Rectum | 59 (22.3%) | 48 (38.1%) |  |
| Missing | 5 (1.9%) | 2 (1.6%) |  |
| TNM Stage |  |  | <0.001 |
| I | 109 (41.1%) | 24 (19.0%) |  |
| II | 54 (20.4%) | 30 (23.8%) |  |
| III | 72 (27.2%) | 41 (32.5%) |  |
| IV | 28 (10.6%) | 24 (19.0%) |  |
| Missing | 2 (0.8%) | 7 (5.6%) |  |
| TNM Stage |  |  | 0.003 |
| I-II | 163 (61.5%) | 54 (42.9%) |  |
| III-IV | 100 (37.7%) | 65 (51.6%) |  |
| Missing | 2 (0.8%) | 7 (5.6%) |  |
| Death for CRC |  |  | 0.017 |
| No | 224 (84.5%) | 93 (73.8%) |  |
| Yes | 41 (15.5%) | 33 (26.2%) |  |

gFOBT: guaiac fecal occult blood test; CRC: colorectal cancer

**Figure legend**

**S1 Fig. Multivariate analyses for colorectal cancer mortality in gFOBT participants**

1. **Interval cancer defined as those diagnosed ≤24 months.**

^a^Hazard ratios adjusted by the variables shown in this figure.

CRC: colorectal cancer

**
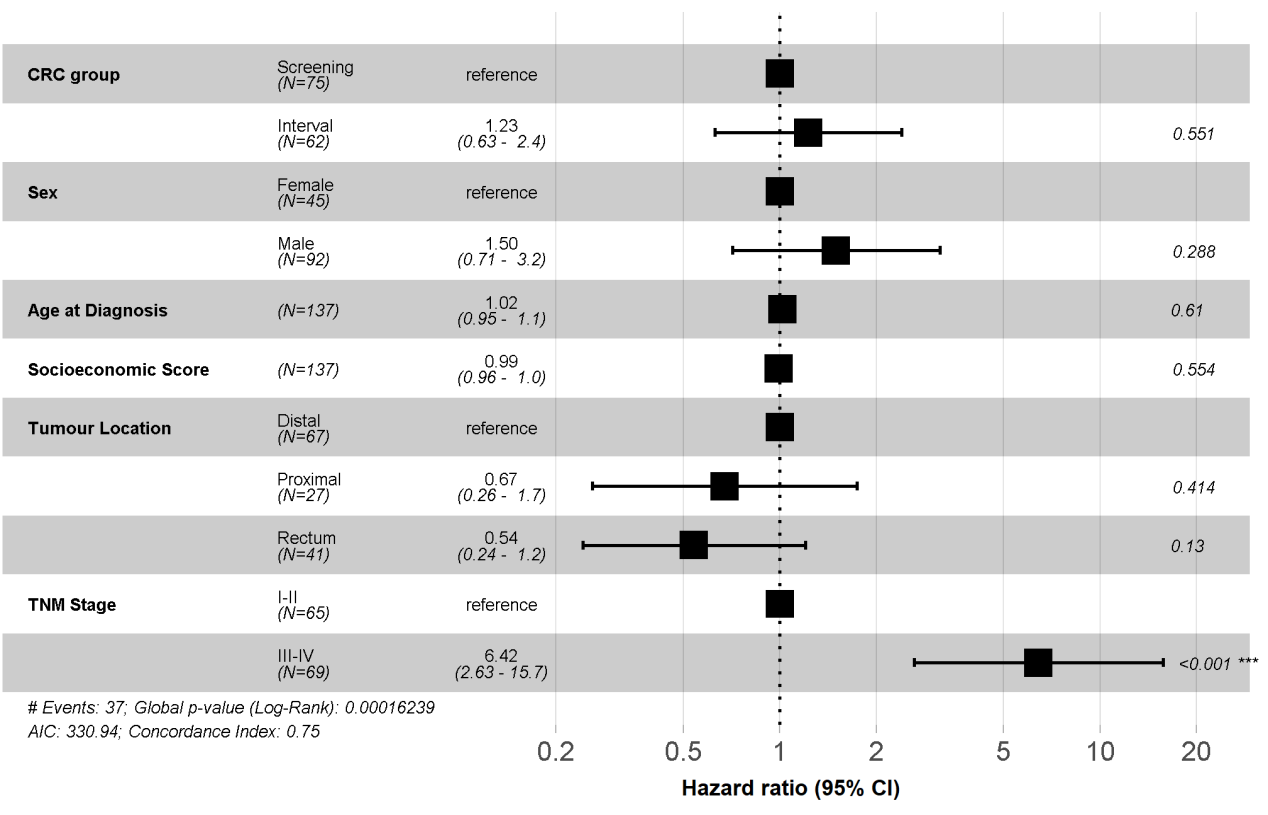
**

1. **Interval cancer defined as those diagnosed ≤30 months.**

^a^Hazard ratios adjusted by the variables shown in this figure.

CRC: colorectal cancer

**
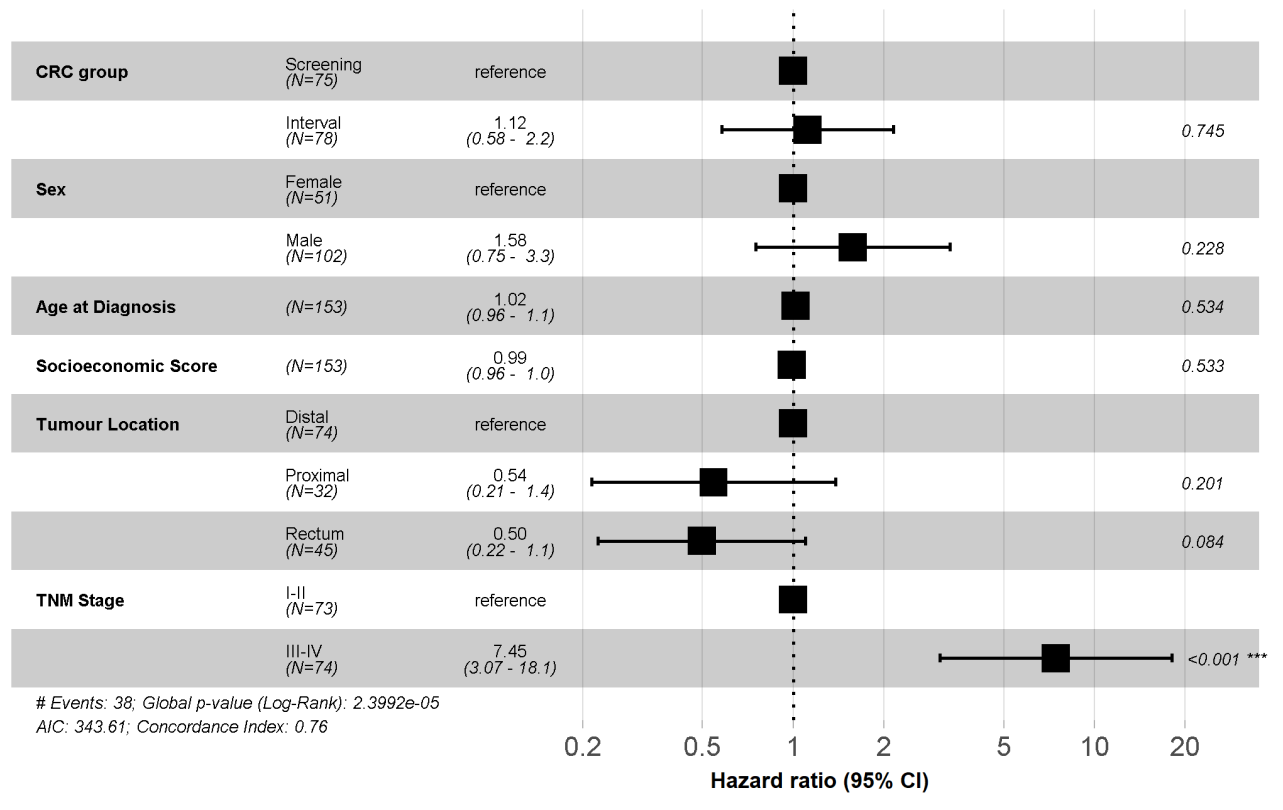
**

**S2 Fig. Kaplan–Meier curves of screen-detected cancers and interval cancers stratified for early (TNM stage I-II) and advanced (TNM stage III-IV).**


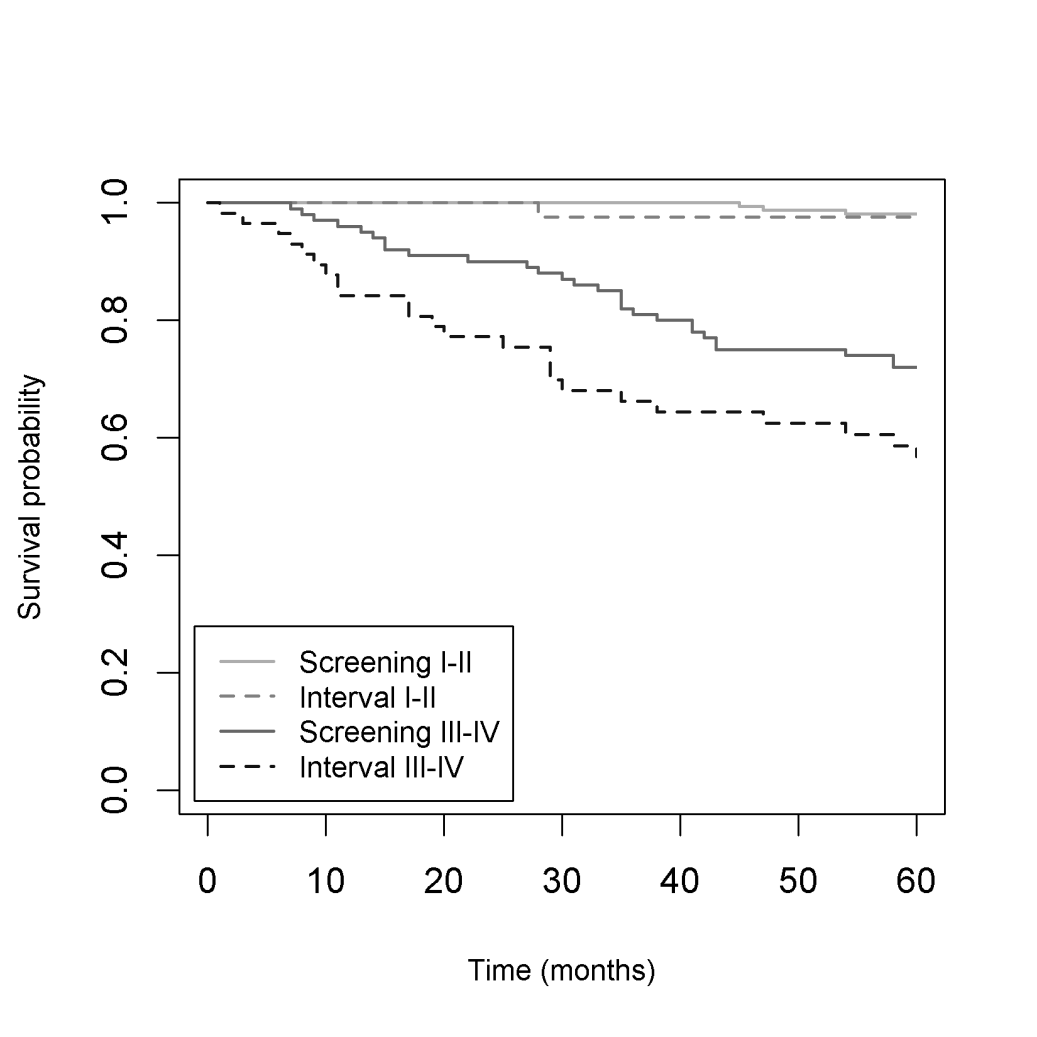

Supplement: S1 File — (DOCX) [file pone.0253369.s001.docx]
